# Clinical research and leadership training program as a knowledge translation initiative across an Australian health care service

**DOI:** 10.1186/1472-6963-14-S2-P78

**Published:** 2014-07-07

**Authors:** Marie Misso, Dragan Ilic, Alison Hutchinson, Terry Haines, Christine East, Helena Teede

**Affiliations:** 1 Monash Centre for Health Research and Implementation - MCHRI, School of Public Health and Preventive Medicine, Monash University in partnership with Monash Health, Melbourne, Australia; 2 Department of Epidemiology and Preventive Medicine, School of Public Health and Preventive Medicine, Monash University, Melbourne, Australia; 3 Centre for Nursing Research, Deakin University and Monash Health Partnership, Melbourne, Australia; 4 Allied Health Research Unit, Monash Health, Melbourne, Australia; 5 Southern Physiotherapy Clinical School, Physiotherapy Department, Monash University, Melbourne, Australia; 6 Maternity Services, Monash Health, Melbourne, Australia; 7 Diabetes and Vascular Medicine Unit, Monash Health, Melbourne, Australia

## 

Health professionals need to be integrated more effectively in clinical research initiatives to ensure that research addresses key clinical needs and provides practical, implementable solutions at the coal face of care. The recent McKeon review of Health and Medical Research in Australia suggested that the best way to achieve practical and implementable solutions in healthcare is to involve the health-delivery workforce in research to ensure that research addresses key clinical needs [1]. Through a partnership with Monash Health and Monash University, goals of the Monash Centre for Health Research and Implementation (MCHRI) are to deliver clinical research and leadership training to build capacity in implementation and support clinical research at Monash Health. Here we describe the informative phase of a broader program to enable and support health professionals at Monash Health who do not have a research background, to engage in and lead research to improve healthcare outcomes. As one of the largest health services in Australia, comprising more than 40 community services and hospitals across Melbourne, Monash Health provides the ideal setting. The broader program is based on a framework that has been shown to lead to successful implementation and long-term sustainability and draws upon knowledge translation principles, as well as medical education frameworks. The study design incorporates mixed methods within a collaborative action research approach involving multidisciplinary researchers across MCHRI, Monash Health and Monash University. Ethics approval has been obtained and literature review completed. Within the informative phase, an online survey and semi-structured interviews are being conducted to explore knowledge, perceptions, experience and training preferences in clinical research methods, evidence based principles and research leadership. We anticipate information from a representative group of people working across different areas and at different levels at Monash Health. The findings will be used to develop a dedicated clinical research and leadership training program. The training program will support Monash Health staff to up-skill or enhance skills to conduct rigorous research; engage and lead multidisciplinary, collaborative teams; and to use research to guide practice, as well as identify and address gaps in clinical research.
